# Stem cell nebulization therapy for COVID-19 infection: radiological and clinical outcomes

**DOI:** 10.1186/s43055-021-00492-3

**Published:** 2021-04-28

**Authors:** Gina M. Torres Zambrano, Yasmine Maher Ahmed, Yendry Ventura Carmenate, Momena Essam Elsadawy

**Affiliations:** Ain Shams University, Cairo, Egypt

**Keywords:** COVID-19, Radiological findings, Chest X-ray, Chest computed tomography, Stem cell nebulization

## Abstract

**Background:**

SARS-CoV-2 is a highly contagious virus that spread worldwide with a rapid increase in the number of deaths. In March 2020, the WHO declared SARS-CoV-2 a pandemic. The primary diagnostic test is reverse transcription-polymerase chain reaction, but chest X-ray and computed tomography have become the typical diagnostic tools used to detect abnormal lung changes. Within the framework of the SENTAD COVID Study clinical trial, an observational and analytical study was conducted, where the patients received nebulization therapy with the use of autologous stem cells (group A) compared to the control arm (group B). Both groups received the UAE SARS-CoV-2 standard management. Radiological images of each patient were collected within 24 h of inclusion in the trial and during follow-up. Herein, we describe and evaluate the radiological findings and outcomes.

**Results:**

A total of 139 subjects were included in our analysis, 69 in group A and 70 in group B. The most common finding was ground glass opacifications, followed by patchy consolidations, with 20% normal radiological images scored 3 from admission until discharge (*p* < 0.0001). Our results suggest a significant improvement in radiological images after treatment secondary to the stem cell effect of reducing inflammation and stimulating the pneumological healing process.

**Conclusions:**

The use of novel therapies, such as stem cells, shows efficacy not only in terms of the control of clinical and paraclinical signs but also in the radiological changes described in the disease.

**Trial registration:**

Study evaluating the safety and efficacy of autologous non-hematopoietic peripheral blood stem cells in COVID-19. Trial registration number: NCT04473170. Date of registration: 16 July 20202. Retrospectively registered.

## Background

In December 2019, severe acute respiratory syndrome coronavirus 2 (SARS-CoV-2) appeared in Wuhan, China. In March 2020, the World Health Organization (WHO) declared SARS-CoV-2 a pandemic. SARS-CoV-2 is a highly contagious virus that spread worldwide with a rapid increase in the number of deaths. It is characterized by fever, sore throat, body aches, dry cough, and dyspnoea. Globally, as of 14 December 2020, 70.829.855 cases had been confirmed, including 1.605.091 (2.22%) deaths reported to the WHO. In the United Arab Emirates (UAE) from the 3rd of January to the 14th of December 2020, 184.949 cases were confirmed, with 617 deaths (0.33%) [1, 2].

Reverse transcription-polymerase chain reaction (RT-PCR) is the most widely used diagnostic test due to its high sensitivity (between 56 and 83%) and the low probability of false positives, but the predictive value of the test varies over time, having a 100% false negative rate at the time of exposure, which decreases at symptom onset from 20 to 38% [3–5]. Among the paraclinical findings, a normal or decreased total WBC count and lymphopenia are found, and elevated liver enzymes, LDH, and C-reactive protein are negative prognostic factors. In critically ill patients, the value of D-dimer increases, which is associated with a multi-organ imbalance [6–8].

Many studies have reported the clinical characteristics of SARS-CoV-2 and abnormalities found in chest imaging, where chest computed tomography (CT) and chest radiography (CXR) have become routine diagnostic tools used to detect abnormal lung changes [6, 9–11].

The role of these images in the detection and diagnosis of SARS-CoV-2 is still debatable. The latest American College of Radiology (ACR) criteria should be considered, taking into account that the findings are nonspecific and overlap with other viral infections, such as hemagglutinin type 1 and neuraminidase type 1 (H1N1), Middle East respiratory syndrome (MERS), and severe acute respiratory syndrome (SARS). The first-line imaging investigation for the diagnosis and follow-up of suspected and confirmed SARS-CoV-2 cases is CXR, which itself is not diagnostic but will help with clinical suspicion while awaiting PCR results [10, 12].

The most common radiological findings described include ground glass opacities (GGOs), patchy consolidation, air bronchogram, crazy pavement pattern, linear opacities and thickening or distortion of the bronchial wall, pleural effusion, and lymphadenopathy. Furthermore, it is essential to keep in mind that there is a relatively common number of normal radiological studies, particularly in early-stage or asymptomatic cases [11].

Using autologous peripheral blood non-hematopoietic enriched stem cells (PBNHESCs), the research team at the Abu Dhabi Stem Cell Center (ADSCC) developed a supportive treatment for SARS-CoV-2 (clinical trial of the “SENTAD COVID Study”) [13].

In this article, we analyzed the radiological changes observed in CXR and chest CT performed in the two groups, one after receiving treatment and the other after being recruited as a control.

## Objective

The aim of this study was to describe the radiological findings and outcomes in patients with SARS-CoV-2 before and after intervention within the framework of the ADSCC SENTAD COVID clinical trial in the Emirate of Abu Dhabi during April 2020.

## Methods

**Fig. 1:**
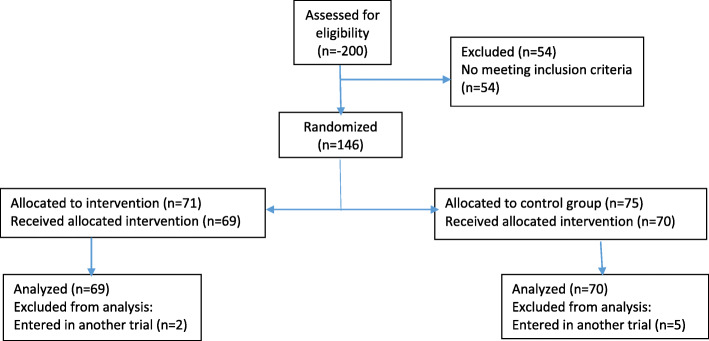
CONSORT flow diagram

Within the framework of the SENTAD COVID Study clinical trial [13], we performed a multi-center, prospective, analytical study to determine the radiological findings and outcomes in patients after the intervention. Four different hospitals from the Emirate of Abu Dhabi participated: Sheikh Khalifa Medical City, Al Rahba Hospital, Al Mafraq Hospital, and Al Ain Hospital. From the main study, a total of 139 patients were divided into 2 groups for comparison (Fig. 1):

1. Group A (experimental arm) SARS-CoV-2 standard treatment plus nebulization with PBNHESCs (*n* = 69)
2. Group B (no intervention arm) received standard SARS-CoV-2 treatment (*n* = 70)

Both groups met the following inclusion criteria: aged ≥ 18 years, laboratory confirmed SARS-CoV-2 infection, interstitial lung change in computed tomography, hospitalized and symptomatic patients, referring one or more symptoms, ability to complete blood collection and test requirements, and agreement to participate in the study.

Patients meeting any of the following criteria were excluded: a diagnosis of any type of shock, organ transplantation in the past 3 months, current immunosuppressive therapy, a diagnosis of hepatitis B virus (HBV) infection, human immunodeficiency virus (HIV) infection or acquired immunodeficiency syndrome (AIDS), a current diagnosis of cancer or a history of malignancies in the past 5 years, pregnancy or lactation, participation in other clinical trials in the past 3 months, an inability to complete blood collection and test requirements, or an inability to provide informed consent.

### Randomization

The study registration/enrolment procedure is found in the data registration system. Fifty patients were expected for each group if there was no resizing of the sample. Randomization was performed to assign the SENTAD-COVID study participants to maintain a balance between the treatment groups. Each “block” had a specific number of randomized treatment assignments. For example, for study groups A and B, the ADSCC research team planned to enrol 10 new patients per week.

Patients were assigned a treatment based on a specified randomized option for the week, having five As and five Bs within each block, so that at the end of each week there was a balance between the two arms of treatment. If the clinical trial had been completed, e.g., after having enrolled 54 patients, it might not be an exact balance, but it would be close.

All demographic characteristics, vital signs, biochemical studies, and radiological images of each patient were collected within 24 h of inclusion in the trial. The WHO ordinal scale for SARSCoV-2 from 1 to 8 was used to determine the clinical severity of the illness (Table 1) [14].

**Table 1:** SENTADO-COVID study seven-category ordinal scale for clinical involvement

Category	Score
No limitation of activities, discharged from hospital	1
Limitation of activities.	2
Hospitalized, no oxygen therapy.	3
Oxygen by mask or nasal prongs.	4
Non-invasive ventilation or high-flow oxygen	5
Intubation and mechanical ventilation.	6
Mechanical ventilation + additional organ support: ECMO, CRRT, vasopressors.	7
Death	8

*ECMO* extracorporeal membrane oxygenation, *CRRT* continuous renal replacement therapy

Both groups received standard SARS-CoV-2 treatment following the “UAE National Guidelines for Clinical Management and Treatment of SARS-CoV-2, v.2.0” according to the Department of Health (DOH) [2]. For group A patients, therapy with autologous stem cells PBNHESC-C (cocktail rich in very small embryonic stem cells (VSEL) and growth factors derived from platelet-rich plasma (commercially called UAE Cell 19) was administered in two nebulizations of 10 cc. In two consecutive days (at least 22 h between each sample collection), withdrawal of 300 cc of whole blood was performed and processed at the ADSCC laboratory, with characterization of cells by flow cytometry and automated inverted fluorescence microscopy.

Chest X-ray was performed for 100 cases (32 from group A and 68 from group B) after confirming respiratory signs using a Samsung GF50 X-ray unit in ADSCC, portable GE definition Amx 700 and fixed GE discovery 650 in Sheikh Khalifa Medical Center. Forty CT scans (37 from group A and 3 from group B) were performed according to the critical criteria indicated using GE 46 MSCT. Both groups were followed up until discharge, and control radiological image reports were taken for further analysis.

### Statistical analysis

A non-normal distribution of the variables was found, so non-parametric statistical methods were used. A test of comparison of proportions (chi-square) was used for the percentage incidence of radiological findings, and the Mann-Whitney *U* test was used for the date ranges. A significance level of *p* < 0.05 was adopted.

The study was approved by the Emirates Institutional Review Board for COVID-19 Research (ID Ref: DOH/CVDC/2020/1172). In accordance with the Declaration of Helsinki, study participants provided written informed consent (World Medical Association Declaration of Helsinki: Ethical Principles for Human Medical Research [Internet]. Vol. 310, JAMA-Journal of the American Medical Association. JAMA; 2013 [cited 2020 Oct 18]. P. 2191–4. Available from: https://pubmed.ncbi.nlm.nih.gov/24141714/) [15]. In the informed consent document, the importance of participation was highlighted and the characteristics of the study and the possible risks and benefits were explained. The patients signed the consent with full knowledge of it, as well as its risks and benefits; additionally, they approved the disclosure of laboratory results and images, as long as the protection of personal data was ensured. All data were kept confidential and the identity of the participants was unlinked. Additional data is available by sending an email to the corresponding author. The selection of diagnostic tools followed the ethical principles of maximum benefit and non-maleficence. This manuscript has the approval of the ethical committee concerning publishing data derived from the main clinical trial. Our study adheres to CONSORT guidelines.

## Results

A total of 139 patients were included in the study: 129 (93%) males and 10 (7%) females total, with 65 (94%) males and 4 (6%) females in group A and 64 (91%) males and 6 (9%) females in group B. The ages of group A ranged from 27 to 71 years (mean 45.9 years old) and from 26 to 73 years old in group B (mean 44.31 years old), with no significant differences between the two groups (*p* = 0.3677).

The median hospital stay after receiving the study treatment was 5 days for group A and 6 days for group B (control), but the range for group B was from 2 to 125 days compared to 1 to 43 days for group A (*p* = 0.2924).

**Fig. 2:**
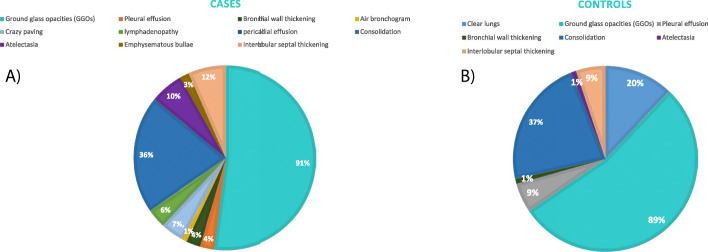
Radiological findings among the studied patients. **a** Patients treated with stem cell nebulization. **b** Control patients

At the time of recruitment, a total of 40 CT scans (37 from group A and 3 from group B) and 100 CXR scans (32 from group A and 68 from group B) were performed. The most common finding was GGOs, appearing in 91 of the cases and 89 of the controls (*p* = 0.6955), followed by consolidations in 25 of the cases and 26 of the controls (*p* = 0.9029), interlobular septal thickening (*p* = 0.5653), and pleural effusion (*p* = 0.2342), with 20% of patients in the control group having normal radiological findings despite being scored 3 from recruitment until discharge (*p* = 0.0001) (Fig. 2).

During follow-up, up to 5 imaging controls were made in group A and 6 in group B. The preferred technology was CXR, while non-contrast chest CT was used in 44% vs CXR in 50% during the second control and 7% vs 93% in the third only in group A. In the first control after treatment or only recruitment, depending on the group, 22% of the reported patients showed favorable improvement in their radiological findings in group A vs 0% in group B (*p* < 0.0001); in the third control, 19% of each group (*p* = 1.0000), in the fourth 40% from group A vs 0% from group B (*p* < 0.0001), and 100% in the fifth control of the remaining patients of each group. Only 6% of group A patients were reported to have lymphadenopathy (*p* = 0.382). GGOs persisted as the most common finding in all control images in both groups, followed by consolidations. No other findings were mentioned by the corresponding radiologist from the different hospitals, such as reticular patterns or pericardial effusion (Table 2).

**Table 2:** Number of radiological images and findings during the follow-up in both arms

Cases	CT (n)	X-Ray (n)	Clear lungs (n)	GGOs (n)	Pleural effusion (n)	Bronchial wall thicke-ning (n)	Air broncho-gram (n)	Crazy paving (n)	Lympha-denopathy (n)	Consolida-tion (n)	Atelectasia (n)	Emphyse-matous bullae (n)	Interlobular septal thickening (n)	Improving (n)
**0**	36	32		63	3	3	1	5	4	25	7	2	8	
**1**	14	16		32	2			1		11	1		3	15
**2**	1	13		13	3	2				9	2		1	6
**3**		10		8	1	1				6	1		1	7
**4**		5		5	2					3				2
**5**		2		1						2				2
**Controls**														
**0**	3	67	14	62	6	1				26	1		6	
**1**		30		27	2	1				19	2	1	2	
**2**		21		19	1	2				13	2		1	4
**3**		7		7						6				6
**4**		2		1						1				
**5**		1		1						1				1
**6**		1		1		1				1				1

*GGOs* ground glass opacities, *0* at the recruitment

**Fig. 3:**
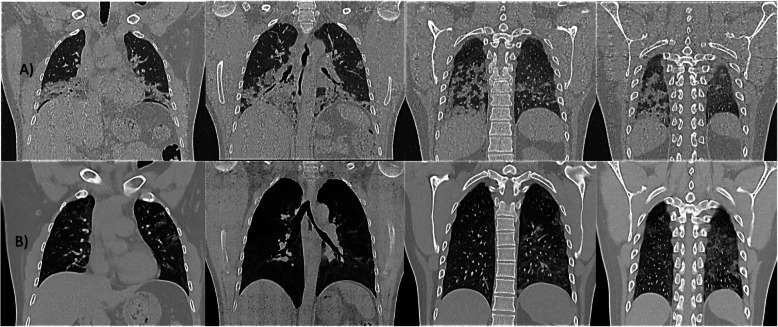
Case 5: A 42-year-old male, without comorbidities, score 4 at the moment of the recruitment, with oxygen requirements through a nasal cannula. High-resolution CT chest: **a** day of admission (April 1st). There are right lung lower lobe and middle lobe extensive consolidations. Similar peripheral opacities noted in the left lung lower lobe and upper lobe. **b** Day 4 after treatment (April 13th, nebulization did on April 9th). Significant improvement of previously noted patchy consolidations at bilateral lower lobes and right middle lobe, as well as few peripheral consolidations in the left upper lobe

**Fig. 4:**
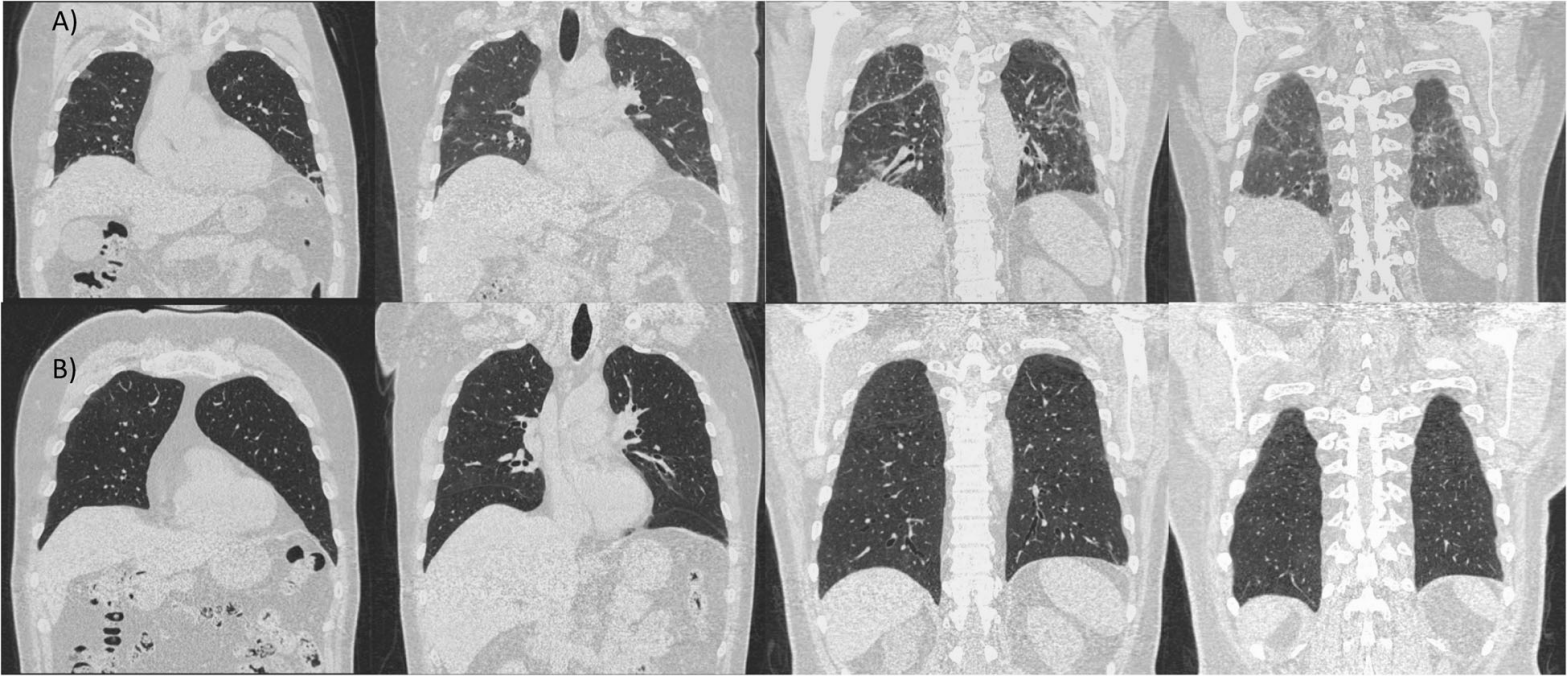
Case 36: 58-year-old male with a history of hypertension, score 3 at the moment of inclusion. CT chest: **a** day of inclusion (April 11th). Bilateral multi-lobar multi-focal, peripheral and subpleural mixed attenuation groundglass, nodular, and linear opacities without crazy-paving pattern noted in bilateral lung parenchyma predominantly involving the bilateral lower lobes. **b** Day 8 after treatment on (April 21st, nebulization did on April 13th). A few scattered areas of ground-glass shadowing in the bilateral lungs. Significant resolution compared to the previous examination

**Fig. 5:**
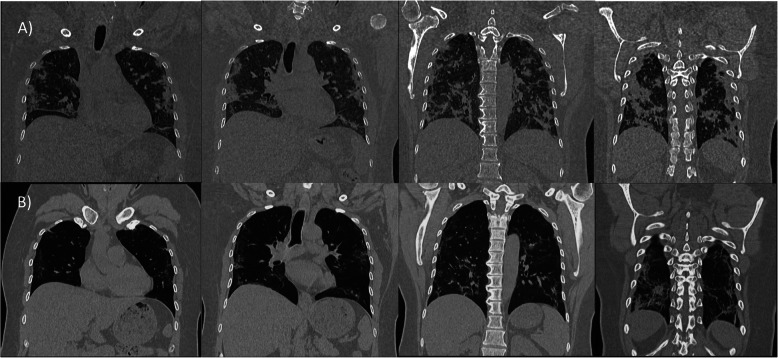
Case 51: 44-year-old female (healthcare worker), with a history of hypertension and hypothyroidism under treatment, score 4. CT chest: **a** day of admission (April 14th). Multi-focal areas of ground-glass opacification/consolidation in the bilateral lungs-lesions. **b** Day 5 after treatment (April 20th). The ground-glass opacities and organized consolidation is seen previously in both lungs in subpleural and peripheral locations appear to be less dense with less size comparing with the previous study done on 14 April 2020 suggestive of good responding to treatment with markedly resolving disease

**Fig. 6:**
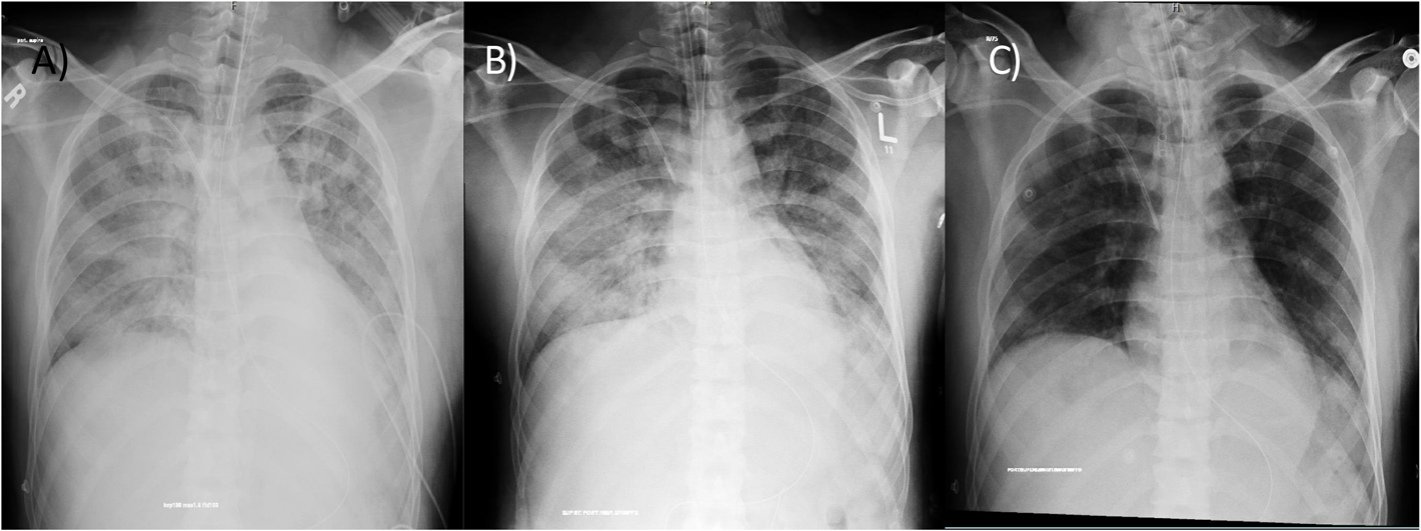
Case 65: a 38-year-old male, with mild obesity, no other comorbidities, score 7 under vasopressor treatment. Chest X-ray: **a** day of inclusion (April 16th). Extensive consolidations involving both lung fields with left retrocardiac volume loss. Blunting of the left CP angle. Ill-defined opacities seen in bilateral lower zones. **b** Day 4 after treatment (April 20th). Improved appearance of patchy airspace opacity in the whole left lung with almost resolved bilateral pleural effusion and underlying atelectasis; however, consolidation is again noted in the left retrocardiac area with mild worsening of airspace opacity in the right mid and lower zones. **c** Day 9 after treatment (April 25th). Regression of patchy airspace opacification mid-right and left lung when compared to the previous study. Residual patchy bilateral airspace opacification particularly in the left retrocardiac region

**Fig. 7:**
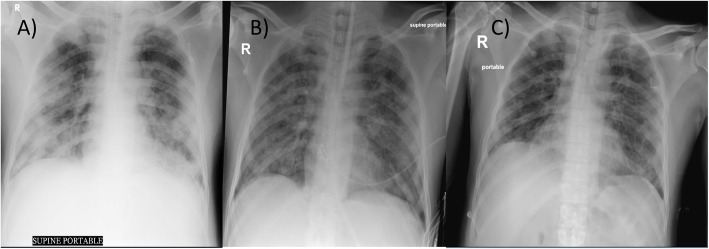
Case 70: 47-year-old male, overweight (BMI 27.88) stayed at the ICU for 14 days, score 7, initially intubated, with post-critical polyneuropathy and grown Staphylococcus epidermidis Isolated from blood culture, resistant to Benzylpenicillin, Clindamycin, Erythromycin, Fusidic Acid, Oxacillin, Trimethoprim/Sulfa. Chest X-ray: **a** day of admission (April 17th). Extensive areas of diffusely organized consolidation throughout both lungs in all lobes, almost involving most of the lower lobes. **b** Day 4 after treatment (April 21st). X-ray partial resolution of the disease noted. **c** Day 13 after treatment (April 30th). Bilateral patchy opacities with mid to lower lung zone predominance as compared with previous partial resolution of the disease noted

We selected relevant images from the treatment group before and after the treatment, which are shown in Figs. 3,4, 5, 6 and 7.

## Discussion

In SARS-CoV-2 patients, a radiological imaging examination is a decisive tool for diagnosis, assessing disease severity and monitoring the clinical course, helping to reduce morbidity and mortality through early diagnosis, suitable treatment, and prevention of disease spread [16].

**Fig. 8:**
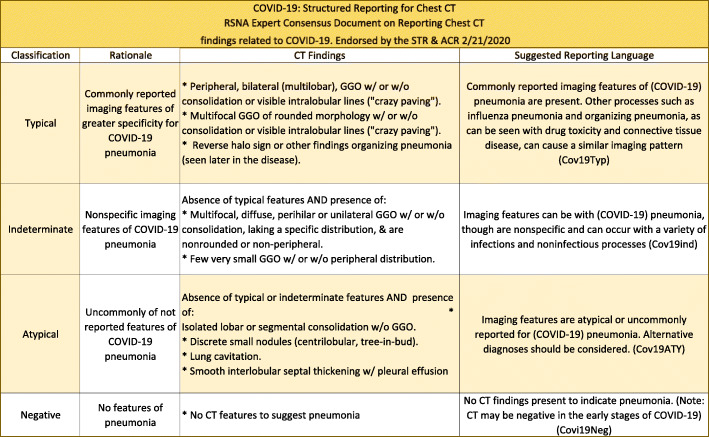
American College of Radiology (ACR) Recommendations for the use of chest radiography and computed tomography (CT) for suspected COVID-19 infection

The RSNA Expert consensus document on reporting chest CT findings related to SARS-CoV-2 endorsed by the STR (Society of Thoracic Radiology) and ACR on 24 March 2020 [12] is shown in Fig. 8.

**Fig. 9:**
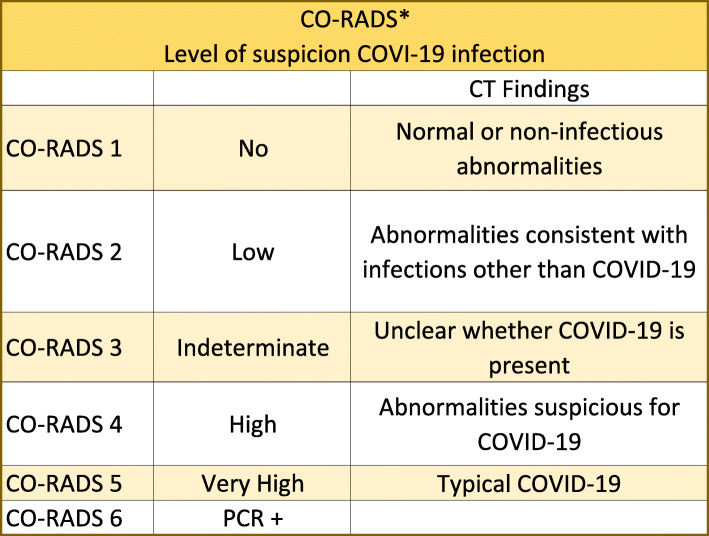
CORADS classification for the level of suspicion of COVID-19 infection based on the CT findings

The level of suspicion for SARS-CoV-2according to the CT findings ranges from very low or CO-RADS 1 to very high or CO-RADS 5, and clinical severity is determined based on comorbidity and differential diagnosis data [17]. CO-RADS 1 has a high negative predictive value in patients with symptoms for four or more days, and CO-RADS 5 has a very high positive predictive value. However, interobserver variation for CO-RADS 2-4 is high and has a negative and poor predictive value. Therefore, interpretation of CT findings should be combined with clinical symptoms and duration, as CT can be negative during the first days of mild infection (Fig. 9).

Mesenchymal stem cells (MSCs) have immunomodulatory effects that can neutralize the intensified immune response and repair damaged and injured cells and tissues in the context of acute respiratory distress syndrome (ARDS). MSCs secrete multiple factors involved in the regeneration and modulation of tissue damage, such as epidermal growth factor (EGF), platelet-derived growth factor (PDGF), fibroblast growth factor (FGF), hepatocyte growth factor (HGF), vascular endothelial growth factor (VEGF) and insulin-like growth factor, and lung injury in vivo [7].

MSCs have been used to treat SARS-CoV-2 to reverse the cytokine storm [18–20]. These cells act in various processes of innate and adaptive immunity, which likely cause changes in the activation of the immune system in patients with inflammatory diseases and by stimulating secretion of anti-apoptotic and regenerative factors.

The number of MSC-based clinical trials in these SARS-CoV-2 subjects is increasing. To date, there are nearly 60 clinical, multi-phase trials evaluating MSC therapy in SARS-CoV-2 subjects listed on clinicaltrials.gov [21]. Most of these trials have not been published, as they are ongoing. According to 1 clinical study of 10 subjects with SARS-CoV-2 pneumonia, 7 were administered MSCs, while the remaining 3 were used as controls [21, 22]. MSC transplantation was safe, with no reports of infusion-related effects or delayed hypersensitivity, and led to reduced serum CRP, the normalization of white blood cell counts, and symptomatic relief of pneumonia.

In this study, we identified a high incidence of GGOs and consolidations without differences between the two groups; corresponding to a meta-analysis, these findings were reported in 94.5% (52/55) of studies [11]. Our results suggest a significant improvement in radiological images in group A after treatment secondary to the stem cell effect on reducing inflammation and stimulating the pulmonological healing process. This method is already being used as a treatment option for lung inflammation via nebulization, with an increase in the number of lungs’ endothelial cells, accelerating airway healing and favorably altering the pathophysiological changes [23].

Few stem cell therapy trials have been reported, most of which were performed by intravenous injection techniques and not by nebulization. Two studies described outcomes of SARS-CoV-2 patients who received umbilical cord-derived MSCs in different regimens. Zhang et al. infused a single dose of 1 million cells/kg in a critically ill SARS-CoV-2 patient who significantly benefited from therapy and was discharged 7 days after the procedure [20]. Liang et al. treated a critically ill SARS-CoV-2 patient unresponsive to prior glucocorticoids, antivirals, and antibiotics with a dose of three 50 million MSCs. No adverse effects were observed, and the patient presented clinical and biomarker improvement after the second cellular infusion [24].

Leng et al. conducted a pilot trial designed in parallel in seven critical patients with SARS-CoV-2 pneumonia treated with intravenous administration of human MSCs and a control group with three patients. Reportedly, treated patients showed relief of all symptoms 2 to 4 days after receiving MSC infusion with no apparent adverse effects. These findings were supported by decreased pneumonia infiltration indicated on chest CT scan and negative results of the SARS-CoV-2 nucleic acid test 2 weeks after infusion [19].

Previously, within the framework of this clinical trial, we examined the frequency of acute kidney injury (ARI) in patients with SARS-CoV-2 and its relationship with clinical outcomes, finding it in approximately one-third of critically ill patients [7]. In addition, those who received the treatment showed a tendency to improve in terms of hospital stay and the evolution of biomarkers (amelioration of lymphopenia [8, 18, 25], neutrophil-lymphocyte ratio [26–29], and C-reactive protein (CRP)) compared to those in the control group. Likewise, we identified stem cells as a protective factor against secondary infection in SARS-CoV-2 cases in terms of sepsis and UTI, also finding an indiscriminate and massive use of antimicrobial agents, which is why rational use of these agents is needed.

The fact that the range in hospital stay after the intervention was much shorter in group A than in group B, given the homogeneity of the treated group thanks to the therapy, and the statistical dispersion of the control group data, although the median was not significant between the groups, may explain why there were fewer radiological controls in the treated group since shorter hospital stay is a protective factor against additional complications and nosocomial infections [7, 30, 31].

Following international recommendations, public hospitals in Abu Dhabi performed most of the radiographic control with CXR [30] due to infection control issues related to transporting patients to CT rooms for SARS-CoV-2 cases. It was also suggested that portable chest radiography should be considered to minimize the risk of cross-infection and in settings with limited access to the reliable real-time reverse transcription-polymerase chain reaction (RT-PCR) test [12]. Additionally, a positive CXR may avoid the need for a CT scan, minimizing radiation exposure.

## Conclusions

In our study, patients in the treatment group exhibited a tendency to improve more rapidly with respect to chest radiological images. CXR itself is not diagnostic but does help with clinical suspicion while awaiting PCR results. The latest ACR appropriateness criteria should be considered, keeping in mind that the findings are non-specific and overlap with other viral infections, such as H1N1, SARS, and MERS. The possible immunomodulatory effect of stem cells offers a therapeutic strategy to control the disease and avoid several associated complications, making it a crucial and potentially promising adjuvant tool for curing and achieving early recovery from SARS-CoV-2, especially in severe infections. The use of novel therapies, such as stem cells, has shown efficacy not only in terms of the control of clinical and paraclinical signs but also in terms of radiographic changes described in the disease; however, larger-scale studies are warranted for further assessment of the radiological correlation with the clinical condition of SARS-CoV-2 patients pre- and post-stem cell administration.

## Data Availability

No data is available due to the patient data protection law.

## Abbreviations

ACR: American College of Radiology
ADSCC: Abu Dhabi Stem Cell Centre
AIDS: Acquired immunodeficiency syndrome
ARDS: Acute respiratory distress syndrome
BMI: Body mass index
CT: Computed tomography
CXR: Chest X-ray
CRRT: Continuous renal replacement therapy
ECMO: Extracorporeal membrane oxygenation
EGF: Epidermal growth factor
DOH: Department of Health
FGF: Fibroblast growth factors
GGOs: Ground glass opacities
H1N1: Hemagglutinin type 1 and neuraminidase type 1
HBV: Hepatitis B virus
HGF: Hepatocyte growth factor
HIV: Human immunodeficiency virus
MERS: Middle East respiratory syndrome
MSCs: Mesenchymal stem cells
SARS: Severe acute respiratory syndrome
SARS-CoV-2: Severe acute respiratory syndrome coronavirus 2
PDGF: Platelet-derived growth factor
PBNHESC: Peripheral blood non-hematopoietic enriched stem cells
RT-PCR: Reverse transcription polymerase chain reaction
UAE: United Arab Emirates
VEGF: Vascular endothelial growth factor
VSELs: Very small embryonic-like stem cells
WHO: World Health Organization
